# EEG-based Quantitative Analysis of Aesthetic Emotion in Clothing Design

**DOI:** 10.1515/tnsci-2019-0008

**Published:** 2019-04-23

**Authors:** Jingjing Wang

**Affiliations:** 1 Zhengzhou Technology and Business University, Zhengzhou 451400, China

**Keywords:** behaviours, EEG analysis, Mood analysis, Fashion design, Aesthetic

## Abstract

This paper combines the perceptual aesthetic sentiments with the objective physiological responses of the human brain. By analysing the characteristics of the brain electrical activities in processing of different types of sentiments, this paper uses the specific physiological indicators of the brain to quantify the aesthetic sentiments about clothing. The purpose is to introduce the EEG quantitative analysis method for sentiment perception into the aesthetic field of clothing, and establish a theory of quantifying aesthetic sentiments towards clothing design through the brain electrical physiological responses.

## 1 Introduction

Sentiment is a complex process integrating multiple components, dimensions, categories and levels. Each occurrence of sentiment is related to physical and psychological factors, instincts and habits, and natural and social factors ^[1]^. From various sentiment theories, it can be seen that sentiment is a mental emotion or feeling that is different from cognition or will. It occurs in a specific situation and is a physical state related to changes in various parts of the body, accompanied by obvious or subtle behaviours.

The aesthetic sentiment of clothing can be summarized as the feeling that a person obtains through visual or tactile sensation in the aesthetic process of the clothing. It is the attitude and experience of the person towards the aesthetic object ^[2]^. Such attitude and experience can lead to positive or negative feelings. Therefore, the generation of clothing sentiment is closely connected with the corresponding aesthetic process.

The traditional way of studying sentiment is usually to describe different sentiment dimensions by designing a semantic scale, which uses degree quantifiers, such as fairly, very, moderately and rarely, to evaluate the degree of sentiment ^[3]^. This method can well track the emotions and feelings of people, but it is relatively subjective. Now that the modern design concept focuses on more objective evidence, it is necessary to convert sentiment, which involves the complex mechanism of psychological processes, into an objective theory and applied technology. For example, Taiwanese scholars use the female vest as an example to use adjectives to evaluate the mood of colour, and get a popular vest colour with fresh, Easy and soft features; or through the “wear acceptance”, “wearing concern” and other aspects to develop clothing interest scales, and obtain support for letter and validity; or use degree quantifiers, such as: very, Very, general, and rarely, etc. to assess the degree of change in mood. These methods can better track people’s emotional feelings, but they are relatively subjective. Under the premise that modern design concepts pay more and more attention to objective evidence, it is necessary to convert emotions, which are complex mechanisms involving psychological processes, into an objective theory and applied technology, and transform emotional sensory quantities into physiological changes.

It is known that people can respond physiologically when they feel various external stimuli and carry out a series of psychological activities. This reaction can be measured by appropriate instruments and means and expressed by a series of physical indicators. Emotion is the result of objective stimulation on the cerebral cortex. Specifically, it is the result of the synergy of the neural processes under the cortex and cortex under the leading role of the cerebral cortex. Therefore, according to the working principle of the human brain when processing different types of emotions, the specific physiological indicators of the brain can be used to quantify the emotions, and the emotional events of the clothing aesthetic emotions are explained from a rational perspective.

From the biological point of view, sentiment is the result of objective stimuli acting on the cerebral cortex, that is to say, it is the result of the synergistic action of the cortex and subcortical nerve processes under the predominant action of the cerebral cortex. Therefore, according to the working principle of the human brain when it is processing different types of sentiments, we can quantify the sentiments using specific physiological indicators of the brain ^[4, 5]^, and explains the aesthetic sentiments towards clothing from a rational perspective.

## 2 Aesthetic sentiments towards clothing design

**Figure 1:**
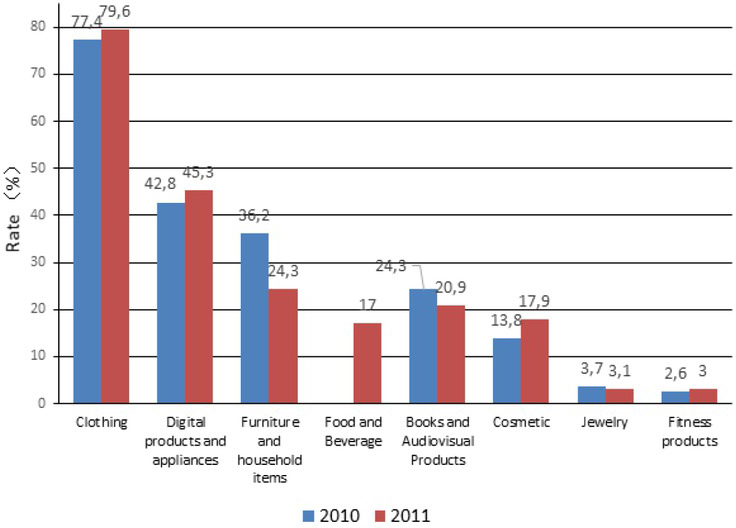
2011-2012 Online Shopping

At present in the clothing market, demand consumption and fashion consumption are changing to personalized consumption, making clothing style an important factor influencing consumers’ choices. Figure 1 shows the online shopping status in 2011 and 2012. It can be seen that clothing accounted for the largest proportion of all consumptions and became the most important part of consumer life.

In addition, with the development of science, people started to study the perceptual sentiments rationally by the quantitative analysis approach ^[6]^. This concept was first proposed by the Japanese scholar Mituo Nagamachi, which is called Kansei engineering. He thought that Kansei engineering is a translation technique that translates customers’ feelings and intentions into design elements. At present, Kansei engineering, design psychology and ergonomics have been widely used in product design, which emphasize emotional design. Therefore, quantifying the aesthetic sentiments with specific physiological indicators according to the brain’s responses and changes in processing of different types of sentiments can help people better understand an individual’s cognitive process.

### 2.1 Product sentiment and research model

Combining perceptual aesthetic emotions with the objective physiological responses of the human brain is by analysing the characteristics of EEG activity when processing different types of emotions, the aesthetic emotions of the clothing are quantified by means of specific physiological indicators of the brain. The purpose is to introduce the EEG analysis method of emotional feeling into the aesthetic field of clothing, and establish the theory that the clothing aesthetic emotion is quantified through the human brain electrophysiological response, and fill the blank of the quantitative analysis of the aesthetic emotion EEG in the clothing field.

As an important category in product design, fashion design is the primary factor in people’s life production. With the continuous improvement of fashion design system, the in-depth development of garment psychology, clothing aesthetics and fashion design ergonomics, the clothing field has begun to pay attention to Rational, systematic, and scientific methods for creating clothing. In addition to requiring the clothing to have the basic functions of traditional cover, comfort, and beauty, it gradually began to pay attention to whether it meets the comprehensive requirements of people, including the physiological and psychological

feelings of human beings. Therefore, using the brain science research method to quantify the aesthetic emotions by using the brain’s specific physiological indicators according to the brain’s reaction and changes in processing different types of emotions, we can understand the individual’s cognitive process to a greater extent. Establishing the theory that clothing aesthetics can be quantified through the human brain electrophysiological response fills the gap in the field of aesthetics for aesthetic emotions.

**Figure 2:**
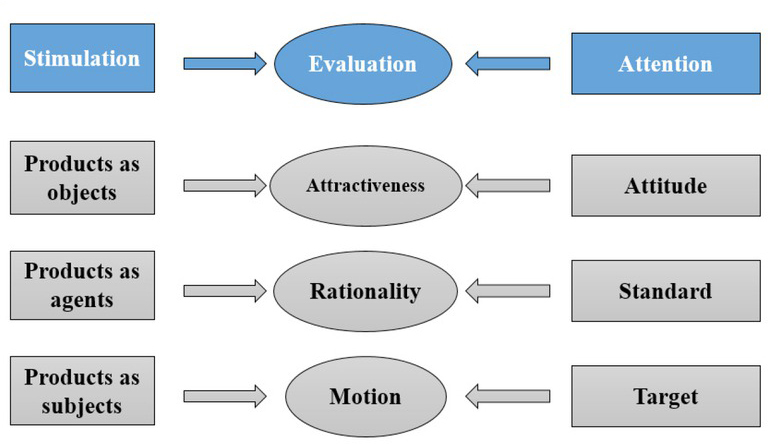
Product sentiment model

The sentiment study is generally based on the individuals’ daily experience. It is generally believed that sentiment is produced under the stimuli from the outside world. According to the study of product sentiment and the product sentiment model, an individual product form can also trigger people’s sentiment responses by stimulating consumers’ evaluation criteria, which provides theoretical basis for the market research on product characteristics ^[7,8]^. Figure 2 shows the widely used product sentiment model, which explains how the product sentiment is produced from a cognitive perspective.

### 2.2 Relations between clothing style and sentiment experience

**Figure 3:**
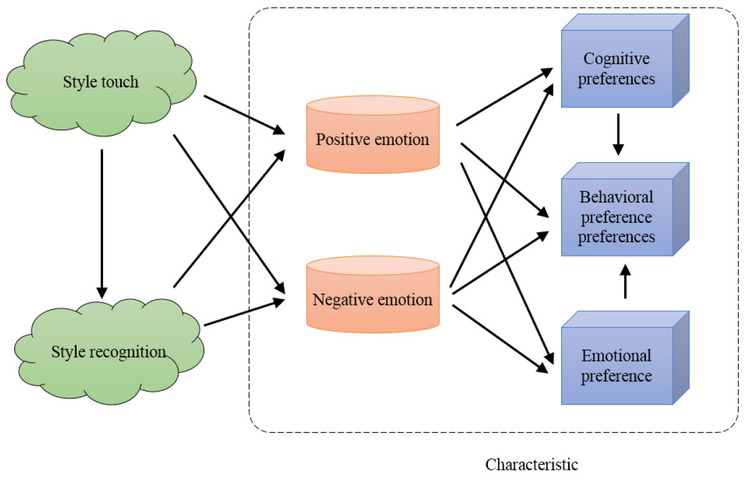
Clothing style preference theory model based on consumer personality and emotion

The word “style” came from the lettering carved on stencils by Romans with needle or pen. The initial meaning was related to the distinctive way of writing. Since then, its meaning has been generalized, not only in literature but also in various fields. Obviously, different styles of clothing will give people different sentiment experience, so the design of clothing will significantly affect people’s sentiments ^[9, 10]^. Both sentiment and personality fall within the scope of consumers’ psychological activities. Foreign literature tends to consider individuality in the study of sentiment and preference. Considering the influences of consumer personalities and sentiments, this paper establishes a theoretical model for clothing style preference, as shown in Figure 3. Based on this, this paper analyses the sentiment experience that the clothing style brings to consumers.

## 3 Reflection of sentiments in EEG signals

### 3.1 Functional division of the brain

**Figure 4:**
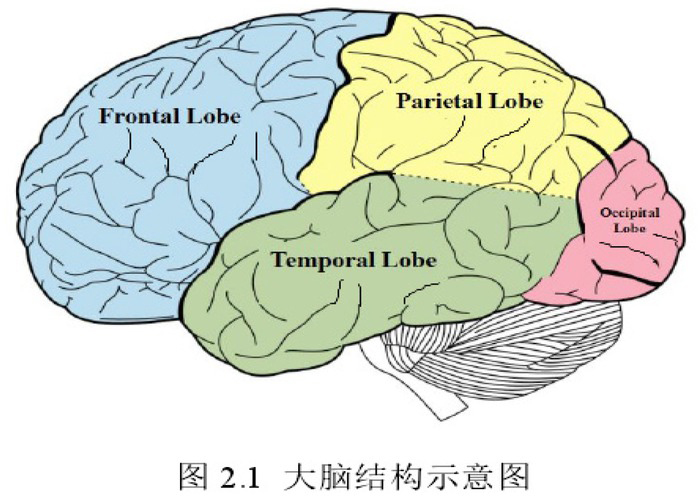
The structure of human brain

The brain is the centre of the nervous system and consists of the left and right cerebral hemispheres. The surface of the cerebral hemisphere is covered with sulci and fissures of varying depths, which divide the cerebral hemisphere into different brain regions, such as the frontal lobe, temporal lobe, parietal lobe, and occipital lobe. Figure 4 shows the four brain regions of the cerebral hemisphere, each of which corresponds to specific functions. It is generally believed that the frontal lobe is related to human’s auto kinetic movements (such as walking) and cognitive functions such as thinking and emotion; the parietal lobe is related to somatic sensation, spatial information and the integrated function of both; the temporal lobe is mainly related to auditory functions; and the occipital lobe is mainly related to visual functions.

Electroencephalography (EEG) is an electrophysiological monitoring method for recording electrical activities in the brain. It can be divided into scalp EEG and cortical EEG ^[8]^. Since the acquisition of cortical EEG data requires a craniotomy of the subject, scalp EEG

is more widely applied in practical applications. For the purpose of this paper, EEG refers to scalp EEG. Scalp EEG is the superimposed reflection of the spontaneous and rhythmic electrical activities of brain neurons on the scalp and is obtained by electrodes placed on the scalp. Figure 2.2 (a) shows the EEG signal with a length of 1s. The frequency range of EEG signals is 0.5-100Hz, and EEG signals with different frequencies have different characteristics. Usually, EEG signals are divided into five frequency bands: delta, theta, alpha, beta and gamma. Its frequency range and characteristics are shown in Table 1.

**Table 1:** The characteristics of different EEG signals

Band name	Range of frequency	Situation	Range of amplitude	Others
Delta	<4Hz	Deep sleep of adults and baby	100uV	
Theta	4~7Hz	Children and sleepy teenagers of adults	20~40uV	
Alpha	7~14Hz	When people are relax	10~40uV	Mainly appear on both sides of the back of the brain. The state of the human brain is often referred to as the subconscious state.
Beta	15~30Hz	Awake state	<30uV	Symmetrically distributed and mainly concentrated in the frontal lobe of the brain
Gamma	30~100Hz	Short-term memory and multi-modality		

### 3.2 Emotion classification model

**Figure 5:**
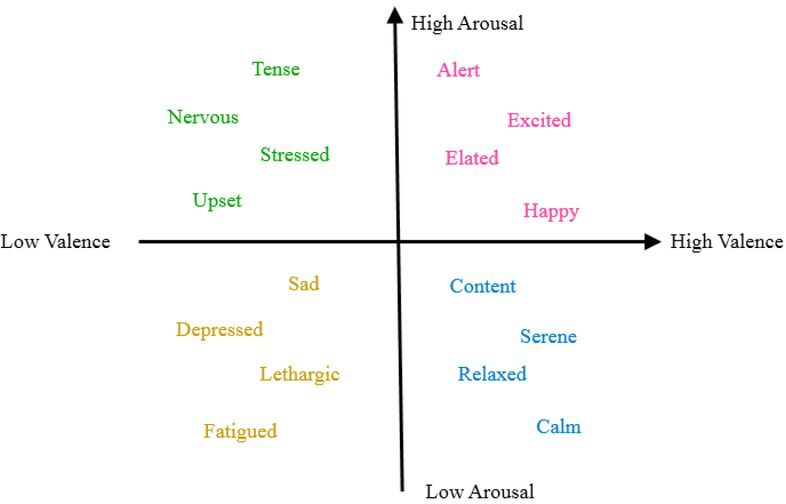
Valence-Arousal model

At present, the study of sentiment recognition mainly focuses on the valence-arousal 2D sentiment model. As shown in Figure 5, in the valence-arousal model, the X-axis indicates the rating of valence, from displeased to pleased, and the Y-axis indicates the degree of arousal, from inactive to active. Different positions in the 2-dimensional space represent different sentiments, and the differences between sentiments can be expressed by the positional differences between each other in the 2D space. For example, “happy” corresponds to pleased and activated, and “sad”, displeased and inactivated.

Successful induction of different sentiment states is a prerequisite for sentiment research. At present, in experiments, visual, auditory or audio-visual stimulus materials with strong emotional colours are often used to induce the subjects to produce different sentiments, and at the same time, relevant response characteristics of the subjects, such as facial expression, EMG and EEG, etc., are recorded. Then the experimental data are compared with pre-recorded data in the experiment to determine the subjects’ sentiments.

## 4 Analysis of aesthetic sentiments towards clothing design with EEG signals

Emotional production is not independent. It is accompanied by changes in the peripheral nervous system, central nervous system, and endocrine system, external behaviour, and psychology. It is a kind of experience, physiology, and interaction that arises from the interaction of objective things with human needs. The integrated psychological process of expressions and these factors interact and influence each other. Although physiological factors such as heart rate, skin electricity, blood pressure, and endocrine changes can provide an effective index of emotional response, the processing of emotions mainly involves the function of the brain. The subjective experience and explicit behaviour of any emotion have a neurophysiological basis, subject to the central nervous system. Changes in mood in the central nervous system can be recorded using relevant instruments.

**Figure 6:**
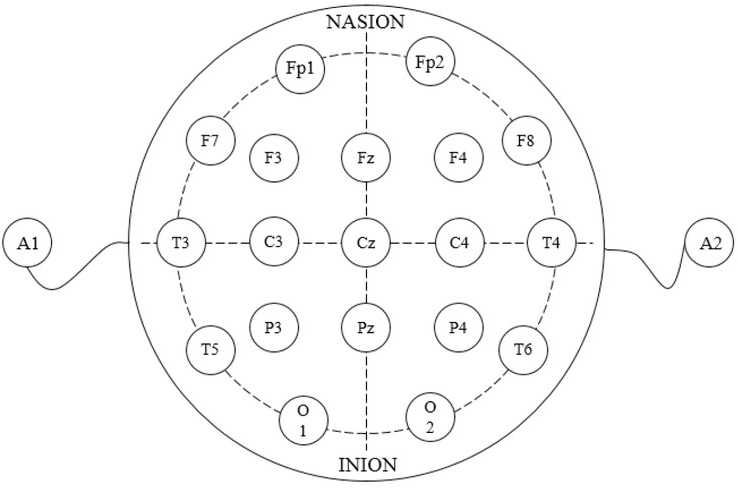
10-20 System Electrode Placements

Obtaining the EEG signals of the subjects under different sentiment states is the

premise of using EEG to identify sentiments. The acquisition of sentiment EEG includes the induction of sentiment EEG and the collection of EEG signals. In this study, the author used strongly stimulating visual and auditory materials to induce sentiments under laboratory conditions and collected and recorded the EEG signals of the subjects, and then showed different styles of clothes to the subjects and recorded their EEG signals. The author compared them with the material signals and in this way obtained the EEG signals stimulated by clothes. The signals were acquired with the electrode cap containing 10-20 system electrodes. The instrument structure is shown in Figure 6.

**Figure 7:**
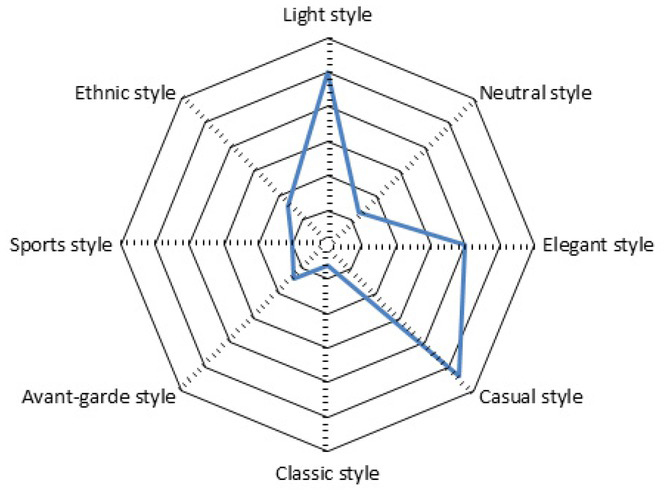
The emotional response of different styles of clothing

By analysing the EEG signals of the subjects, the author obtained the statistics about the degrees of positive sentiments brought by different clothing styles, and generated the map of the subjects’ preferences for different styles, as shown in Figure 7.

The sentiment analysis results obtained from the EEG signals proved to be consistent with the results obtained from subjective questionnaires. Therefore, it is reasonable and effective to use EEG signals to quantify the aesthetic sentiments towards clothing design.

## 5 Conclusions

Because the aesthetic emotion of clothing can be expressed through visual channels or through other sensory channels, such as touch, pressure, temperature, muscle, etc., real-time EEG data can be collected through multi-channel induction during experimental design. In addition, emotions can be evaluated in multiple angles in combination with other physiologically quantified indicators. For example, by combining eye tracker evaluate the positive and negative polarity of emotions by analysing visual attention focus and gaze duration, or adding other neurophysiological reactions and behavioural motives triggered by emotions. And psychological activities such as blood pressure, endocrine changes (including adrenaline, heart rate, blood flow rate, respiration, muscle stretch, gastrointestinal activity, and body temperature related changes) and other physiological indicators or facial colon, expression and other behavioural components and different behaviours Behavioural and so on to specifically analyse the physiological changes accompanying the human body when emotions are generated, and transform the emotional sensory amount into physiological changes, making the results more convincing.

This study integrates physiology, psychology, aesthetics, ergonomics, Kansei engineering, and clothing design and reveals the physiological responses and changes brought about by different sentiments in the aesthetic process of clothing, especially the changes in the brain. It associates physiological signals with psychological activities, combines sentiment self-assessment and event-related potential recordings, and collects electrophysiological evidence of changes in sentiment through EEG measurement. By analysing the changes in the central nervous system with the generation of sentiments, the paper studies the interactions between sentiment dimensions and event-related potentials and establishes the theory of quantifying aesthetic sentiments towards clothing with EEG, which can provide effective guidance to the design and consumption of clothing.
